# First Mexican species of the genus *Cosberella* (Collembola, Hypogastruridae)

**DOI:** 10.3897/zookeys.829.26730

**Published:** 2019-03-11

**Authors:** José G. Palacios-Vargas, Gabriela Castaño-Meneses

**Affiliations:** 1 Ecología y Sistemática de Microartrópodos, Departamento de Ecología y Recursos Naturales, Facultad de Ciencias, UNAM, 04510, México, D. F., México Universidad Nacional Autónoma de México México Mexico; 2 Ecología de Artrópodos en Ambientes Extremos, Unidad Multidisciplinaria de Docencia e Investigación, Facultad de Ciencias, UNAM, Campus Juriquilla, Boulevard Juriquilla 3001, 76230, Querétaro, México Unidad Multidisciplinaria de Docencia e Investigación Querétaro Mexico

**Keywords:** Chaetotaxy, Citlaltépetl, intraspecific variation, taxonomy

## Abstract

A new species of *Cosberella* is described and illustrated from a temperate forest of Citlaltépetl formation, Veracruz State. It is characterized by the following combination of characters: Th I with 2 + 2 dorsal setae; 2 + 2 axial setae on Th II–III; two capitate tenent hairs on each leg; unguiculus half the length of unguis; unguis with tooth; six dental setae and Abd VI without anal spines. A key for the species of the genus is included.

## Introduction

The genus *Cosberella*, was erected by Wray in 1963, and includes several species originally described in the subgenus Mucrella Fjellberg, 1985, and in *Hypogastrura* Bourlet, 1839 or *Achorutes* Templeton, 1835. All of them were transferred to the genus *Cosberella* by Bernard (2006) after a redescription of its type-species. It currently includes eight species distributed in the Holarctic and Nearctic regions. We have found a species new to science in the Neotropical region which is described here.

The abbreviations used in this paper are:

Ant antennal segment;

a anterior row of setae;

Abd abdominal segment;

m median row of setae;

p posterior row of seta.

PAO postantennal organ;

sgd dorsal guard sensillum;

sgv ventral guard sensillum;

Th thoracic segment.

Maxillary lamellae are numbered according to Fjellberg (1984).

## Taxonomy

### Cosberella

Taxon classificationAnimaliaCollembolaHypogastruridae

Wray, 1963

#### Diagnosis.

(after Bernard, 2006) Antenna with or without eversible sac between Ant III and IV. Six to eight sensilla on Ant IV. Ventral sensory file on Ant IV, when present, with 30 or fewer modified setae. Mandible with four or five apical teeth and well-developed molar plate. Maxilla with six lamellae, the fourth one reduced, not exceeding apex of the fifth one. Guard setae of labial palpus pointed; lateral process present or absent. Postantennal organ small, with four small distinct lobes, or simply oval with lobes indistinct; accessory tubercle absent. Unguis with or without tooth, tenent hairs acuminate or clavate. Unguiculus with or without lamella. Pronotum usually with 2 + 2, rarely with 3 + 3 setae. Ventral tube with 4 + 4 or 5 + 5 setae. Tenaculum with 4 + 4 teeth. Dens with six or seven setae, one proximal seta longer than others; mucro with a latero-external lamella of variable shape, its apex tapering, curved. Anal spines minute or absent.

#### Type species.

*Cosberellaconatoa* Wray, 1963.

#### Remarks.

The Mexican species described here is assigned to the genus *Cosberella*. It recalls also the genus *Choreutinula* by the absence of anal spines and a small PAO with four flattened lobes. However, this genus lacks of unguiculus, or has only a short thin bristle in its place, while the unguiculus of the new species is well developed, straight and half as long as claw, and the fourth maxillary lamella is reduced, not exceeding apex of the fifth one, which are characteristics of genus *Cosberella*.

### Cosberella mendozarum sp. n.

Taxon classificationAnimaliaCollembolaHypogastruridae

http://zoobank.org/21837BAF-AF8F-4D66-B0C8-C3191DC0A69B

Figs 1–5 , 6–8

#### Type-locality.

México: Veracruz State (Atotonilco) 7 km from Parque Nacional del Pico de Orizaba. Pine-oak temperate forest, 19°08'30N, 97°12'26W, 2,225 m a.s.l. ex litter from pitfall traps, F. Álvarez leg.

#### Type-specimen.

Holotype male mounted on slide. Original label: “21/2/12 México, Veracruz State, Pico de Orizaba, 2,225 m snm. F. Álvarez col. Pitfall 8” [printed label]. Collection number 22304.

#### Paratypes.

9 paratypes males and 1 juvenile mounted on slides, with the same data. Type material is kept at Colección de Microartrópodos, Facultad de Ciencias, UNAM. Collection numbers 22305–22314.

#### Diagnosis.

Th I with 2 + 2 dorsal setae. Tenent hairs 2, 2, 2 in one whorl. Unguiculus without lamella and half the length of unguis. Unguiculus with a tooth in the apical third. Six dental setae, one proximal longer than others. Anal spines absent.

#### Description.

**Figures 1–5.**
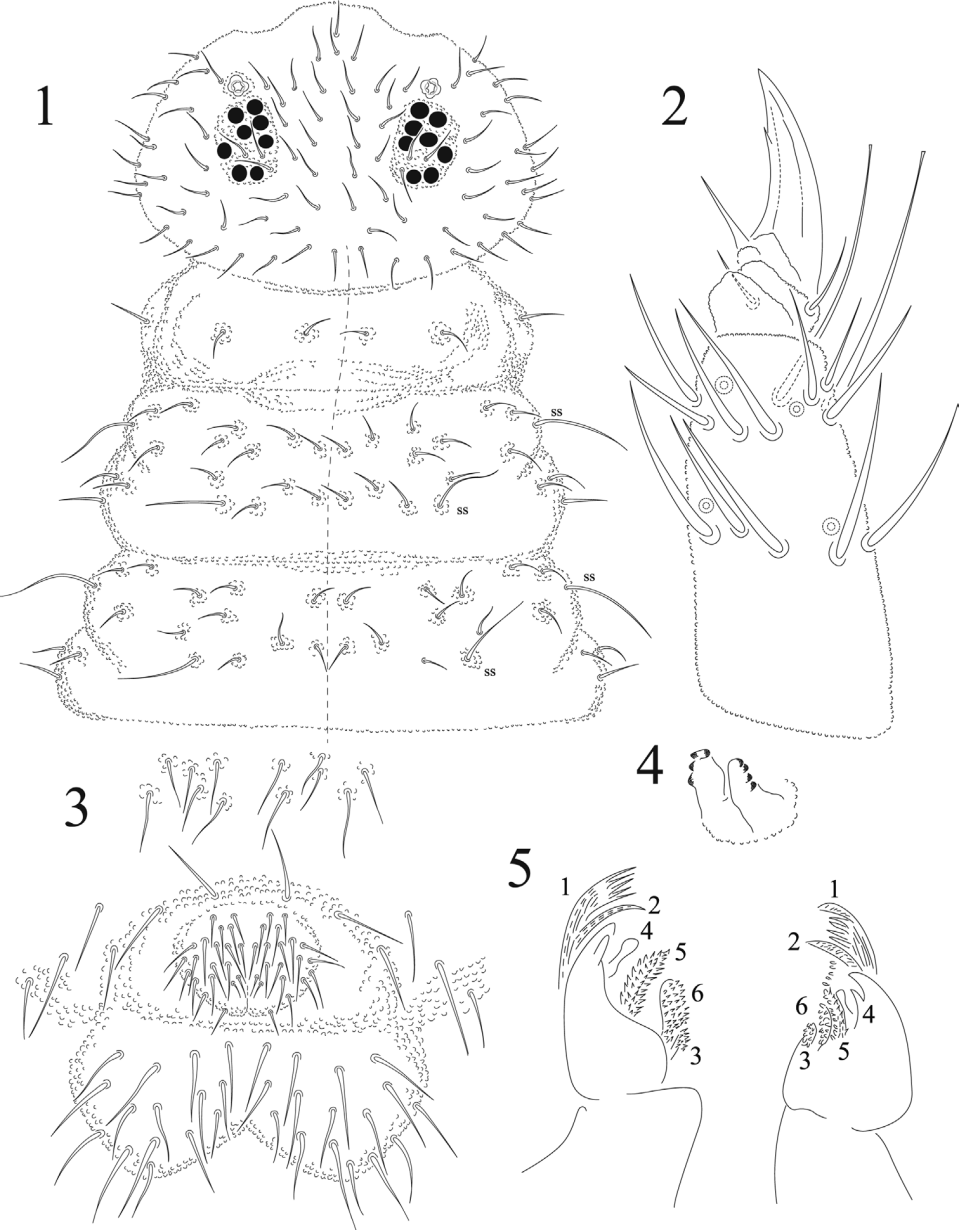
*Cosberellamendozarum* sp. n. **1** head and thorax chaetotaxy **2** tibiotarsus III **3** ventral chaetotaxy of Abd V and VI **4** tenaculum **5** maxillae.

Body length (average of 10 specimens) = 1.06 mm. Setae not differentiated in macro and microsetae, all of same size (Figs 1, 7), smooth and acuminate (6–8 μm). Sensorial setae longer than regular setae (23–25 μm). Sensorial formula as 022/11111. Color light blue in alcohol. Ratio head : antenna 1 : 0.7

**Figures 6–8.**
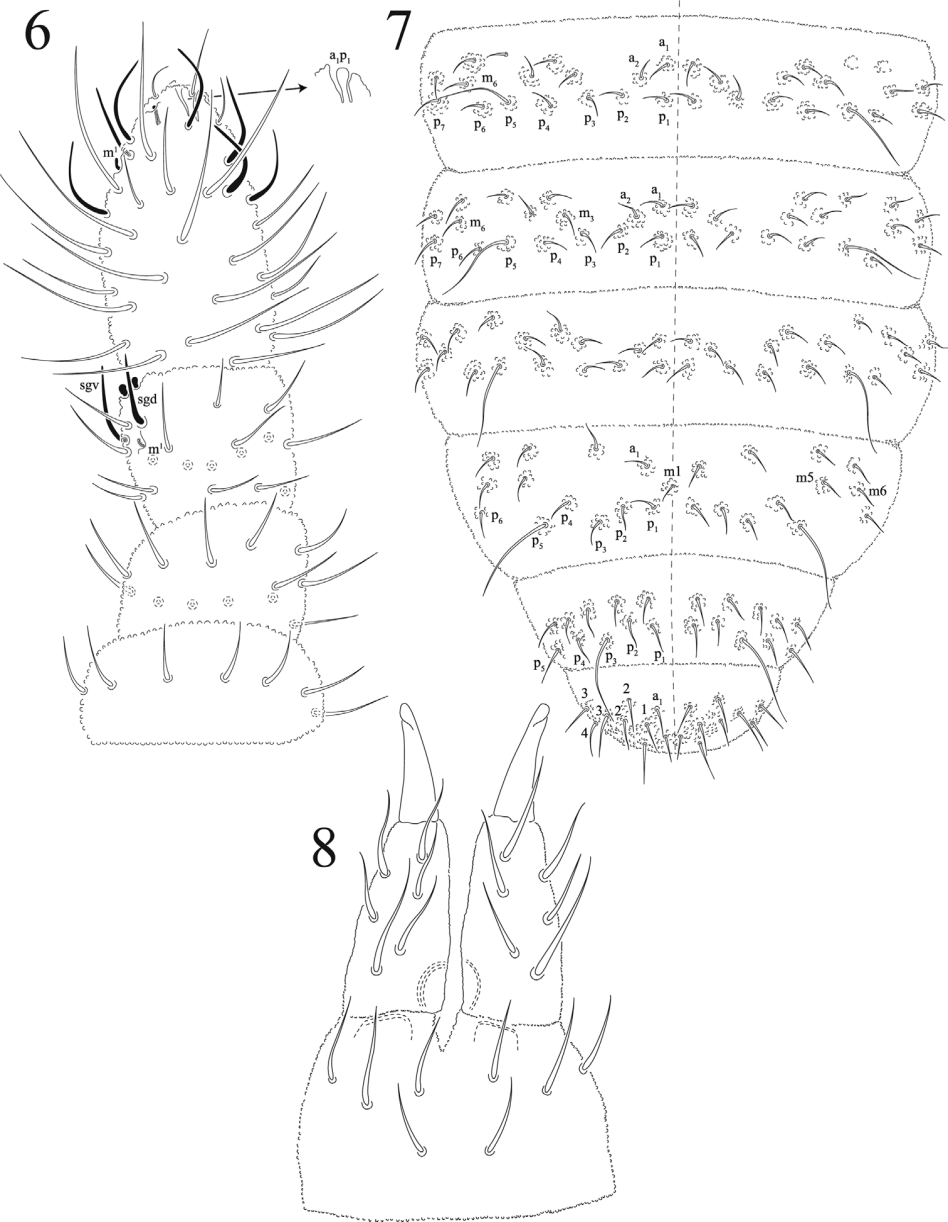
*Cosberellamendozarum* sp. n. **6**Ant I-IV, dorsal view **7** dorsal abdominal chaetotaxy **8** furcula.

Ant I with seven dorsal setae, Ant II with 12 setae. Ant III with 16 setae in two whorls, sense organ with two free club-shaped microsensilla, not covered by tegumentary fold; two long guard sensilla (sgd and sgv) of same shape and size, and one ventral microsensillum. No eversible sac between Ant III-IV. Ant IV with seven sensilla, subapical organite, lateral microsensillum and simple subapical bulb (Fig. 6), no sensory file on ventral side. Ratio Ant I: II; III; IV = 1:1.1; 1.4; 2.5.

Head with almost typical chaetotaxy for the genus but lacking seta c1. Three subequal setae in ocular area (Fig. 1). 8+8 eyes of about equal diameters. PAO made of one vesicle with a small tendency to be quadrangular, as big as closest eye. Labial palpus with six proximal setae; lateral process absent; maxillary palp normal for the genus; two pairs of postlabial setae. Mandible with 5–6 apical teeth, and normal molar plate. Maxilla with six lamellae. Lamella 1 with prominent apical denticles and dorsal minute medial denticles; lamella 2 very thin, ciliate; lamella 3 small, ciliate; lamella 4 reduced, not ciliate; lamella 5 densely ciliate; lamella 6 spherical and ciliate (Fig. 5). Th I with 2 + 2 dorsal setae and 1+1 lateral ones on upper subcoxae. Sensorial setae on Th II and III are m7 and p4 as usual. Each thoracic segment with 3 irregular rows of setae (Fig. 1) with m3 and m5 present; a2 present on Th II but lacking on Th III.

Leg chaetotaxy from I to III: coxae 5,5,7; trochanters 5,5,5; femora 12,11,10 with one ventral seta very long, as acuminate tenent hair; tibiotarsi 19,19,18 (Fig. 2); pretarsi 2,2,2. Two tenent hairs from slightly to clearly clavate on the dorso-distal whorl of each tibiotarsus. Unguis thick, curving slightly, with one ventral tooth in distal third (Fig. 2). Unguiculus straight, acuminate without any lamella. Ratio tibiotarsus/unguis = 1: 0.5.

Ventral tube with 4 + 4 or 5 + 5 setae. Tenaculum with 3–4+3–4 teeth, without seta on corpus (Fig. 4). Furcula well developed. Manubrium with ten pairs of setae, one of them longer. Dens dorsally with moderate granulation and with six setae, one basal longer than others, ventrally with a smooth triangular area less than ¼ of dens length. Mucro half-length of dens, not spoon-like, long and narrow with one very low outer lamella (not illustrated), apex curved (Fig. 8).

Chaetotaxy of abdomen as in Fig. 7. Abd I - III with three irregular rows of dorsal setae, one sensorial seta at P5, except on Abd V where it is at P3. Number of axial setae from Abd I to III is 2 + 2. Abd IV with 3 + 3 such setae but half of the specimens lack one seta (as illustrated on Fig. 7). Abd VI with three rows of setae, a1–3, m1–4 pl-2, p0 displaced posteriorly. No anal spines. Genital plate of male with 3 + 3 pregenital, 38–44 circumgenital and 4 + 4 eugenital setae (Fig. 3). Each anal valve with 15 regular + 3 hr setae. Female unknown.

#### Etymology.

The new species is named after professors Concepción and Enrique Mendoza from the high school “6”, of the UNAM, “Antonio Caso”, for their assistance provided to the senior author during his studies.

#### Discussion.

The new species has an isolated position in the genus due to the combination of a number of characters, some of which are observed in other genera of Hypogastruridae, particularly in the genus *Choreutinula* (thoracic chaetotaxy, number of tibiotarsal tenent hairs, lanceolate unguiculus, complete absence of AS). Nevertheless, the structure of maxillary head with a partial reduction of the fourth lamella (main diagnostic trait of the genus *Cosberella*) unequivocally points to this genus as a better choice.

The new species is most similar to *C.arborea* (Fjellberg, 1992), the only other known species of the genus lacking anal spines. *Cosberellamendozarum* sp. n. can be easily distinguished by its specific chaetotaxy, first of all by the absence of cephalic seta c1, the presence of only 2+2 axial setae on Th II–III (vs 3+3 setae in *C.arborea*), the number of tibiotarsal tenent hairs on mid-leg (2 vs. 3 in two different whorls in *C.arborea*), the number of dental setae (6 subequal vs. 7 of different size in *C.arborea*), and the setae as microsetae, contrary to *C.arborea* which has one macroseta on each side of head and laterally (p6) on each abdominal segment from I to IV. *Cosberellaconatoa* (Wray, 1963) differs from the new species by its minute anal spines, the absence of ungual tooth, different number of tenent hairs and the unguiculus with a strong lamella.

#### Variations.

Ten specimens were studied. They present several setal asymmetries dorsally on abdomen. Few also have duplicated setae in one abdominal alveolus, and in a couple of cases one or two bifid setae on each alveolus of abdominal segments. Two specimens had 2 + 1 postlabial setae (instead of 2 + 2); PAO sometimes had 5 vesicles with a central part in circle, looking as a small flower. In four specimens retinaculum had 3 + 3 teeth instead of 4 + 4. Setae m1 on Abd IV vary asymmetrically, with a single seta on right or left side (normal axial number is 3 + 3). One specimen subadult had the posterior row of setae on Abd V with left side lacking sensorial setae, p1 and p2. Ventral tube had 5 + 5 setae (4 specimens), 4 + 4 (3 specimens), 5 + 6 (1 specimen), 5 + 3 (1 specimen) and 5 + 0 (1 specimen).

### Key to species of *Cosberella*

**Table:** 

1	With true and well developed anal spines	**2**
–	Without anal spines or very weakly developed	**7**
2	Anal spines about 2/3 inner edge of hind unguis (in non-ecomorphic specimens)	**3**
–	Anal spines no more than ½ inner unguis	**4**
3	Anal spines much longer than basal papilla. Ant IV ventral file with 20 – 30 setae (France)	***C.acuminata* (Cassagnau, 1952)**
–	Anal spines about as long as basal papillae. Ant IV ventral file with 10 setae (Tuva, Siberia)	***C.yoshiana* (Babenko, 2000)**
4	Ant IV apical bulb trilobed, ventral file with about 30 setae (Tennessee, USA)	***C.lamaralexanderi* Bernard, 2006**
–	Ant IV apical bulb simple to trilobed, ventral field with less than 16 setae	**5**
5	Body setae long, macrochetae strongly developed. Abd IV with p_1_ twice the length of p_2_ (northern Russia)	***C.navicularis* (Schött, 1893)**
–	Body setae short, most of them microsetae. Abd IV with p_1_ less than twice as long as p_2_	**6**
6	Maxillary lam 6 covered with denticles. Ventroapical hyaline field of dens short, only 1/4–1/3 of dens (Alaska, USA)	***C.denali* (Fjellberg, 1985)**
–	Maxillary lam 6 with 3–4 apical hooks only. Ventroapical hyaline area large, occupying approx. 1/2 of dens (Indiana, USA)	***C.hibernica* (Fjellberg, 1987)**
7	Unguiculus about 2/3 as long as unguis, with broad basal lamella	**8**
–	Unguiculus about 1/2 as long as unguis, straight, acuminate without any lamella (Veracruz, México)	***C.mendozarum* sp. n.**
8	Tibiotarsi without tenent hairs, minute anal spines (Tennessee, USA)	***C.conatoa* (Wray, 1963)**
–	Tibiotarsi with 2–3–2 tenent hairs (Vancouver, Canada)	***C.arborea* (Fjellberg, 1992)**

## Supplementary Material

XML Treatment for Cosberella

XML Treatment for Cosberella mendozarum
